# Human Cerebral Cortex Proteome of Fragile X-Associated Tremor/Ataxia Syndrome

**DOI:** 10.3389/fmolb.2020.600840

**Published:** 2021-01-29

**Authors:** Katharine Nichole Holm, Anthony W. Herren, Sandra L. Taylor, Jamie L. Randol, Kyoungmi Kim, Glenda Espinal, Verónica Martínez-Cerdeño, Isaac N. Pessah, Randi J. Hagerman, Paul J. Hagerman

**Affiliations:** ^1^Department of Biochemistry and Molecular Medicine, University of California Davis School of Medicine, Davis, CA, United States; ^2^Mass Spectrometry Research Core, University of California Davis, Davis, CA, United States; ^3^Department of Public Health Sciences, Division of Biostatistics, University of California Davis School of Medicine, Davis, CA, United States; ^4^Medical Investigation of Neurodevelopmental Disorders Institute, University of California Davis School of Medicine, Davis, CA, United States; ^5^Department of Pathology and Laboratory Medicine, University of California Davis School of Medicine, Davis, CA, United States; ^6^Department of Molecular Biosciences, University of California Davis School of Veterinary Medicine, Davis, CA, United States; ^7^Department of Pediatrics, University of California Davis School of Medicine, Davis, CA, United States

**Keywords:** FXTAS, DIA-MS, SUMO1/2, Tenascin-C, CD38, Fragile X Syndrome, FMRpolyG, FMR1

## Abstract

**Background:** Fragile X-associated tremor/ataxia syndrome (FXTAS) is an adult-onset neurodegenerative disorder associated with premutation CGG-repeat expansions (55–200 repeats) in the 5′ non-coding portion of the *fragile X mental retardation 1* (*FMR1*) gene. Core features of FXTAS include progressive tremor/ataxia, cognitive decline, variable brain volume loss, and white matter disease. The principal histopathological feature of FXTAS is the presence of central nervous system (CNS) and non-CNS intranuclear inclusions.

**Objective:** To further elucidate the molecular underpinnings of FXTAS through the proteomic characterization of human FXTAS cortexes.

**Results:** Proteomic analysis of FXTAS brain cortical tissue (*n* = 8) identified minor differences in protein abundance compared to control brains (*n* = 6). Significant differences in FXTAS relative to control brain predominantly involved decreased abundance of proteins, with the greatest decreases observed for tenascin-C (TNC), cluster of differentiation 38 (CD38), and phosphoserine aminotransferase 1 (PSAT1); proteins typically increased in other neurodegenerative diseases. Proteins with the greatest increased abundance include potentially novel neurodegeneration-related proteins and small ubiquitin-like modifier 1/2 (SUMO1/2). The FMRpolyG peptide, proposed in models of FXTAS pathogenesis but only identified in trace amounts in the earlier study of FXTAS inclusions, was not identified in any of the FXTAS or control brains in the current study.

**Discussion:** The observed proteomic shifts, while generally relatively modest, do show a bias toward decreased protein abundance with FXTAS. Such shifts in protein abundance also suggest altered RNA binding as well as loss of cell–cell adhesion/structural integrity. Unlike other neurodegenerative diseases, the proteome of end-stage FXTAS does not suggest a strong inflammation-mediated degenerative response.

## Introduction

Fragile X-associated tremor/ataxia-associated syndrome (FXTAS) is an adult-onset neurodegenerative disorder caused by premutation (PM) range expansion (50–200 repeats) of the trinucleotide (CGG) repeat element in the 5′ untranslated region of the *fragile X mental retardation 1* (*FMR1*) gene. Approximately 30–40% of male and 8–16% of female PM carriers will develop FXTAS, with onset of initial symptoms typically beginning in males in their early 60's (Leehey et al., 2007; Hagerman and Hagerman, 2016). Symptoms of FXTAS include intention tremor, gait ataxia, parkinsonism, neuropathy, white matter disease, and cognitive decline (Hall et al., 2014; Hagerman and Hagerman, 2016; Kong et al., 2017; Cabal-herrera et al., 2020). The principal neuropathological feature of FXTAS is the presence of generally solitary intranuclear inclusions in both neurons and astrocytes within the central nervous system (CNS) (Greco et al., 2002, 2006; Garcia-Arocena et al., 2009; Martínez Cerdeño et al., 2018), as well as in diverse non-CNS tissues (Greco et al., 2007; Hunsaker et al., 2011), mitochondrial dysfunction (Ross-Inta et al., 2010; Napoli et al., 2011; Kaplan et al., 2012; Cabal-herrera et al., 2020), microglia activation and senescence (Martínez Cerdeño et al., 2018), iron deposition (Ariza et al., 2018), and dysregulation of neuronal Ca^2+^ (Robin et al., 2017; Hagerman et al., 2018). While the correlation between repeat length and cellular dysfunction is well-characterized, the pathway of neuronal dysfunction, age-related disease progression, and incomplete penetrance of FXTAS within the PM population remains generally unresolved.

Several mechanisms of FXTAS pathogenesis have been proposed, many of which are based on increased *FMR1* mRNA expression and protein sequestration. In the RNA toxicity model, the expanded RNA CGG repeat is thought to be responsible for sequestration of proteins such as DiGeorge syndrome chromosomal (or critical) region 8 (DGCR8), thereby decreasing their cellular abundance and function (Norman et al., 2010; Hoem et al., 2011; Qurashi et al., 2011; Pretto et al., 2013; Hagerman and Hagerman, 2016; Sellier et al., 2017; Rodriguez and Todd, 2019). Another proposed, posttranscriptional model involves Repeat-Associated Non-AUG (RAN) translation that is initiated upstream of the AUG start codon, leading to translation through the CGG repeat and resulting in a polyglycine-containing peptide (FMRpolyG). Studies of the possible role of FMRpolyG in FXTAS pathogenesis, generally involving transgenic mouse models and/or *in vitro* cell models, have presented evidence of cellular toxicity of FMRpolyG and colocalization of the peptide with intranuclear inclusions (Todd et al., 2013; Buijsen et al., 2014; Oh et al., 2015; Sellier et al., 2017; Krans et al., 2019; Friedman-Gohas et al., 2020). However, to our knowledge, native FMRpolyG has not been quantified *in vivo* in patient-derived cells, except in trace amounts in the intranuclear inclusions of *postmortem* FXTAS cases (Ma et al., 2019).

Elucidation of differential protein translation and intranuclear protein abundance in PM subjects is essential to profiling the FXTAS proteomic landscape and may provide novel insights into disease pathology. To date, proteomic characterization of end-stage FXTAS in *postmortem* human brain tissue has not been performed. Our current findings suggest that abnormal or ineffective elimination of protein aggregates may underlie FXTAS pathogenesis; however, FMRpolyG, proposed as a driver in co-aggregation models of FXTAS (Todd et al., 2013; Oh et al., 2015; Krans et al., 2019), was not identified in either control or FXTAS *postmortem* brains.

## Materials and Methods

### Study Design/Subjects

Samples of frozen brain tissue from frontoparietal cerebral cortex were obtained from our University of California (UC) Davis FXTAS/FXS Brain Repository and were utilized for the proteomic studies (Table 1). All samples were originally obtained under approved UC Davis Institutional Human Subjects guidelines. A total of eight PM/FXTAS and six control male samples were included in the mass spectrometry (MS) analysis. All PM/FXTAS subjects suffered from end-stage FXTAS, characterized by at least stage 4 and usually stage 6 FXTAS prior to death.

**Table 1:** Subject demographics (males)^a^.

		**Controls**	**Premutation/FXTAS**
		**(*n* = 6, Male)**	**(*n* = 8, Male)**
Age (years) at death		69 + 10.6	82 ± 4.6
PMI (h)		6.5 ± 3	11.5 ± 4.2
CGG repeat length		25 + 8.1	104.1 ± 13.1
**Group**	**CGG repeat size**^***b***^	**Age at death**	**Publication**^***c***^**, case no**.
Control	29	53	
Control	13	69	
Control	25	79	
Control	26	66	
Control	37	N/A	
Control	33	69	
Premutation	93 ± 12	85	
Premutation	110 ± 13	87	a, case 11; b, case 4; c, case 2
Premutation	125	81	
Premutation	88 ± 1	82	c, case 12
Premutation	108 ± 10	78	c, case 16
Premutation	84 ± 13	79	
Premutation	110 ± 15	75	a, case 6; b, case 6; c, case 1
Premutation	93 ± 8	81	a, case 9; b, case 9; c, case 13

a *PMI data are available for three controls and three FXTAS cases only*.

b *For premutation repeats, “±” indicates range of detectable bands for multiple bands*.

c *a, (Greco et al., 2006); b, (Garcia-Arocena et al., 2009); c, (Pretto et al., 2013)*.

*FXTAS, fragile X-associated tremor/ataxia syndrome; PMI, postmortem interval*.

### Sample Preparation

For proteomic analysis, tissue samples were extracted in 1 ml of sodium dodecyl sulfate (SDS) solubilization buffer [5% SDS, 50 mM triethylammonium bicarbonate (TEAB), 1× PhosSTOP phosphatase, and 1× cOmplete Mini Protease Inhibitor tabs (Roche)] and further disrupted by bead beating with a MagNA lyser (Roche) using three rounds of 20 s each at 7,000 rpm. Samples were clarified by centrifugation at 15,000×g for 10 min, and the resulting supernatant was taken for analysis. For each sample, protein concentration was determined by bicinchoninic acid (BCA) assay (Thermo-Pierce) and 100 μg of total protein was volume normalized, reduced and alkylated, and enzymatically digested with trypsin using S-Trap mini (Protifi) spin columns according to manufacturer instructions with the following modifications: samples were reduced with 20 mM dithiothreitol (DTT) (Sigma) for 20 min at 50°C, alkylated with 40 mM indole-3-acetic acid (IAA) (Sigma) for 30 min at room temperature, and digested with two rounds of trypsin (Worthington) addition each at a 1:25 (enzyme:protein) weight ratio. Samples were reacted with the first trypsin round for 2 h at 37°C, followed by a second addition and incubation overnight at 37°C. Samples were eluted, lyophilized (Labconco), and reconstituted in 100 mM TEAB. Peptide concentration was measured by fluorescent peptide assay (Pierce). Each sample was reconstituted in 2% acetonitrile/0.1% trifluoroacetic acid (TFA), and 1 μg was injected for analysis. Equal portions of all samples were mixed together to make a reference sample to be run multiple times for chromatogram library runs.

### Liquid Chromatography Tandem Mass Spectrometry

Digested peptides were analyzed on a Thermo Scientific Fusion Lumos Orbitrap Mass Spectrometer in conjunction with an UltiMate 3000 RSLCnano ultra high-performance liquid chromatography (UHPLC) and EASY-Spray source operating in positive ionization mode. Peptides were loaded on a Thermo Scientific Acclaim PepMap 100 C18 reversed-phase pre-column (100 μm × 20 mm, 100Å, 5U) before being separated using an EASY-Spray C18 reversed-phase analytical column (ES802, 75 μm × 250 mm, 100Å, 2U). Peptides were eluted with an increasing percentage of acetonitrile over the course of a 120-min gradient with a flow rate of 200 nl/min at 40°C.

### Chromatogram Library Creation and Data-Independent Acquisition

Six gas-phase fractionated (GFP) chromatogram library injections were made using staggered 4-Da isolation windows across the mass range 400–1,000 m/z: GFP1 = 400–500 m/z, GFP2 = 500–600 m/z, GFP3 = 600–700 m/z, GFP4 = 700–800 m/z, GFP5 = 800–900 m/z, and GFP6 = 900–1,000 m/z. Targeted MS/MS were acquired in the orbitrap with a higher collisional dissociation energy of 30%, resolution of 30 K, maximum injection time of 60 ms, and an automatic gain control (AGC) target of 800%. Each individual sample was also similarly run in data-independent acquisition (DIA) mode (Hu et al., 2016; Zhang et al., 2020) using staggered isolation windows of 8 Da across the full mass range of 400–1,000 m/z with higher collisional dissociation energy of 30%, orbitrap resolution of 15 K, maximum injection time of 20 ms, and an AGC target of 800%.

### Data Processing and Analysis

DIA data were analyzed using Scaffold DIA v.1.3.1 (Proteome Software, Portland, OR, USA). Raw data files were converted to mzML format using ProteoWizard v.3.0.11748. The Reference Spectral Library was created by EncyclopeDIA v.0.9.2. Chromatogram library samples were individually searched against Prosit predicted databases created using the Prosit online server (https://www.proteomicsdb.org/prosit/) and converted for ScaffoldDIA using the EncyclopeDIA tool. The input for the Prosit prediction consisted of the UniProt human reference proteome with a peptide mass tolerance of 10.0 ppm and a fragment mass tolerance of 10.0 ppm. Variable modification considered included oxidation of methionine, and static modification included carbamidomethyl of cysteine. The digestion enzyme was assumed to be trypsin with a maximum of one missed cleavage site allowed. Peptides identified in each search were filtered by a percolator to achieve a maximum false discovery rate (FDR) of 0.01. Individual search results were combined, and peptides were again filtered to an FDR threshold of 0.01 for inclusion in the reference library.

Peptide quantification was performed within Scaffold DIA. For each peptide, the five highest quality fragment ions were selected for quantitation. Proteins that contained similar peptides and could not be differentiated based on MS/MS analysis were grouped to satisfy the principles of parsimony. Only proteins with a minimum of two identified peptides were considered and filtered by a protein FDR threshold of 1.0%.

### Statistical Analysis

Ion counts were obtained for 6,076 proteins. Protein quantities in each sample were total count normalized by scaling counts to the average sum of counts across all subjects. Proteins with more than two missing values in an experimental group were excluded from statistical analysis, leaving 5,863 proteins for analysis after correction. For each protein, individual values more than 2.5 standard deviations above the mean of log_2_ transformed counts were considered outliers and dropped from the analysis. This resulted in 121 values (0.15%) dropped, but did not result in any protein having more than two missing values per experimental group. Missing values were imputed as one half of the protein-specific observed minimum value. Protein counts were log_2_ transformed for statistical analysis to meet model assumptions. Age at death for one control subject was missing and imputed as the average age of control subjects.

Differential protein accumulation between FXTAS and control groups was first evaluated using two-sample *t*-tests with unequal variances. Second, to account for age difference, linear regression was used to identify differentially abundant proteins (DAPs) between groups with age included as a covariate in the model. For each analysis, Benjamini–Hochberg FDRs were calculated to account for multiple testing. A two-sample *t*-test with unequal variances was used to compare age at death and CGG repeat length between experimental groups. Statistical analyses were conducted in R Statistical Computing Software version 3.6.3. Complete list of proteins is available on Massive (ID: MSV000086400 https://massive.ucsd.edu/ProteoSAFe/dataset.jsp?task=da97eab790e84cefbc13cc3769b0ba64) [activated upon acceptance].

### RNA Extraction

Total RNA was isolated from each 100 mg lyophilized brain sample using Qiazol reagent (catalog # 79306, Qiagen) for tissue lysis and purified using the RNeasy mini kit (catalog # 74104, Qiagen) with column and DNAse treatment. Quality measurements of total RNA were performed using the Agilent Bioanalyzer RNA 6000 Nano kit (catalog # 5067-1511, Agilent Technologies).

### RNA Sequencing

RNA sequencing (RNA-seq) libraries were prepared from the RNA of cortex sections (Table 1). Sequencing and library preparation were performed by the DNA technologies and Expression Analysis Core in the Genome Center of the University of California, Davis. RNA Integrity (RIN) scores were assessed for all samples, resulting in a mean of 6.4 ± 0.87 (range = 4.9–8.3). Gene expression profiling was carried out using a 3′-Tag-RNA-Seq protocol. Barcoded sequencing libraries were prepared using the QuantSeq FWD kit (Lexogen, Vienna, Austria) for multiplexed sequencing according to the recommendations of the manufacturer starting from 300 ng total RNA each. Both the unique dual index (UDI)-adapter and unique molecular identifier (UMI) Second Strand Synthesis modules were used (Lexogen). The fragment size distribution of the libraries was verified *via* microcapillary gel electrophoresis on a LabChip GX system (PerkinElmer, Waltham, MA). The libraries were quantified by fluorometry on a Qubit fluorometer (Life Technologies, Carlsbad, CA) and pooled in equimolar ratios. The library pool was quantified *via* quantitative PCR (qPCR) with a KAPA Library Quantification Kit (Kapa Biosystems/Roche, Basel, Switzerland) on a QuantStudio 5 system (Applied Biosystems, Foster City, CA). The libraries were sequenced on a HiSeq 4000 sequencer (Illumina, San Diego, CA) with single-end 100-bp reads.

### Gene Ontology and Molecular Signatures Database Exploratory Analysis

The Molecular Signatures Database (Subramanian et al., 2005; Liberzon et al., 2016) (MsigDB, v7.1), Gene Set Enrichment Analysis (GSEA), and the Human Protein Atlas (proteinatlas.org, 2000; Thul et al., 2017) was used to evaluate the differences in protein accumulation between controls and FXTAS subjects. Pathway enrichment analysis was conducted to identify enriched groups of proteins that share common molecular functions defined by the C5: Gene Ontology (GO) gene sets, MF: molecular function from MSigDB (https://www.gsea-msigdb.org/gsea/msigdb/annotate.jsp). Only the proteins with a raw *p* < 0.05 (*n* = 423) were included in the enrichment analysis to select overrepresented protein sets. Resulting GO molecular functions were manually characterized into functional families.

## Results

### Demographic Characteristics of Brain Samples Used in the Current Study

Subject characteristics are delineated in Table 1. Proteins used for MS were isolated from frontotemporal cortex of 14 male subjects (eight late-stage FXTAS/PM subjects and six controls without known neurological disease). The average age of FXTAS subjects was 82 ± 4 years (range: 75–87 years) and of control subjects was of 69 ± 10 years (range: 53–79 years). Female cases were not used for these studies to avoid confounding effects of variable activation ratio for the two X chromosomes. As expected, the difference in CGG repeat length between the control and FXTAS group was highly significant (*p* < 0.001), with an average CGG repeat length of 25 (±8.1) in the control group and an average length of 104.1 (±13.1) in the FXTAS group. However, FXTAS patients were also significantly older than the control group (*p* = 0.027). Therefore, age adjustment was performed in the analysis of all proteins for group comparison.

### Identification of Differentially Abundant Proteins Associated With Fragile X-Associated Tremor/Ataxia Syndrome

**Figure 1:**
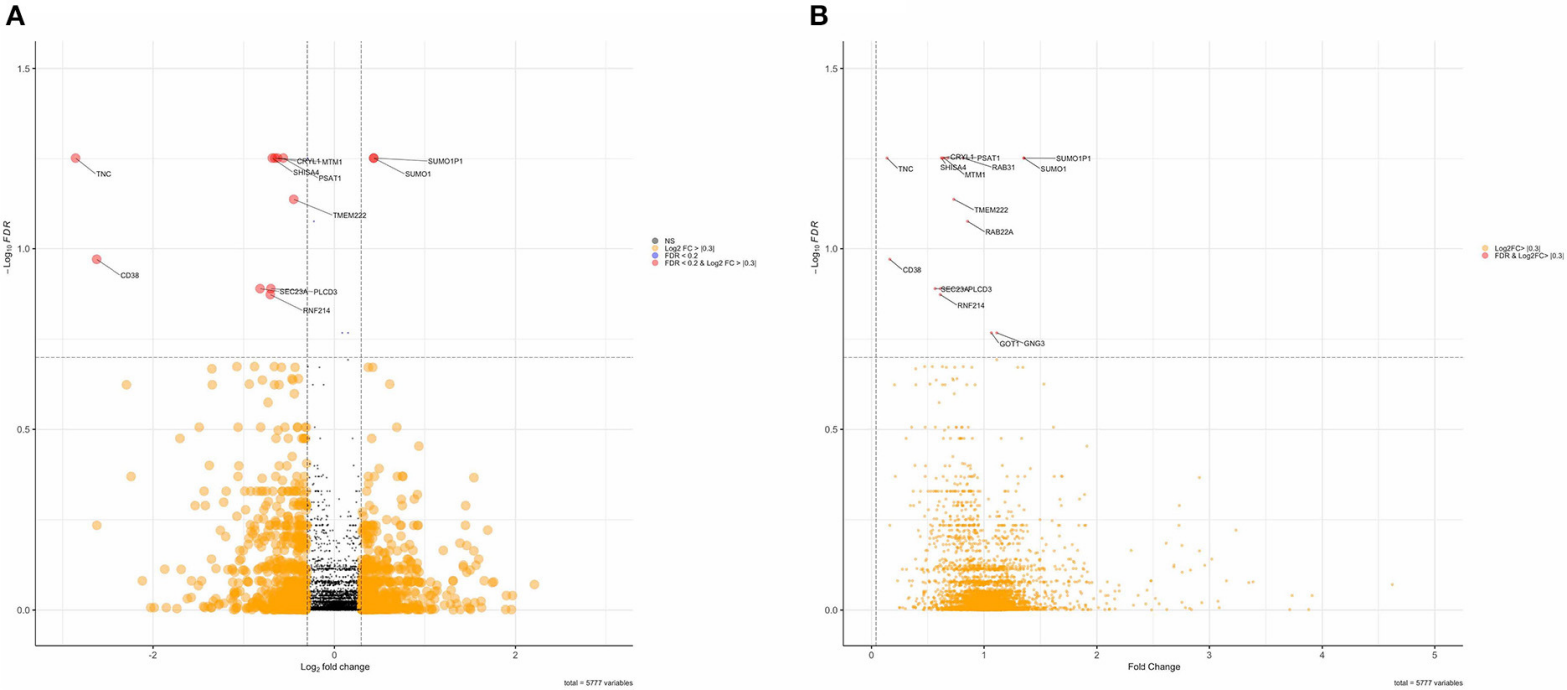
**(A)** Volcano plot of Log2FC and false discovery rate (FDR) values of differentially abundant proteins. Red points are proteins with an FDR < 0.2. **(B)** Volcano plot of fold change values of differentially abundant proteins.

DAPs were determined using two-sample *t*-tests and linear regression to adjust for age differences between FXTAS and control subjects. Without adjusting for age, analysis of the MS data identified 414 DAPs (Table 2) from the initial pool of 5,863 proteins after FDR correction (FDR < 0.2). However, adjusting for age differences reduced the number of DAPs to 16 proteins, of which four proteins demonstrated increased abundance and 12 proteins demonstrated decreased abundance (Table 2). Including age as a covariate did not change the direction of group differences for any of the DAPs but did decrease the magnitude of differences. Overall, the FXTAS cortex proteome does not demonstrate any significant bias toward overexpression or underexpression compared to controls; however, the majority of proteins with significant levels of differential expression are biased toward decreased abundance in FXTAS cortexes. Distribution of fold changes and FDR corrected *p*-values (*q*-values) for all proteins are represented in the volcano plot in Figure 1.

**Table 2:** Differentially expressed proteins (FDR < 0.2).

**Protein (gene symbol)**	**log2FC**	**Raw *p*-value**	**FDR**
TNC	−2.858	0	0.056
CRYL1	−0.657	0	0.056
MTM1	−0.628	0	0.056
PSAT1	−0.564	0	0.056
RAB31	−0.296	0	0.056
SHISA4	−0.685	0	0.056
SUMO1P1	0.437	0	0.056
SUMO1	0.437	0	0.056
TMEM222	−0.449	0	0.073
RAB22A	−0.226	0	0.084
CD38	−2.626	0	0.107
PLCD3	−0.701	0	0.129
SEC23A	−0.82	0	0.129
RNF214	−0.707	0	0.134
GOT1	0.091	0	0.171
GNG3	0.155	0	0.171

*FDR, false discovery rate; PSAT1, phosphoserine aminotransferase 1; RNF214, ring finger protein 214; TNC, tenascin-C*.

### Fragile X-Associated Tremor/Ataxia Syndrome Proteome Pathway Enrichment

**Figure 2:**
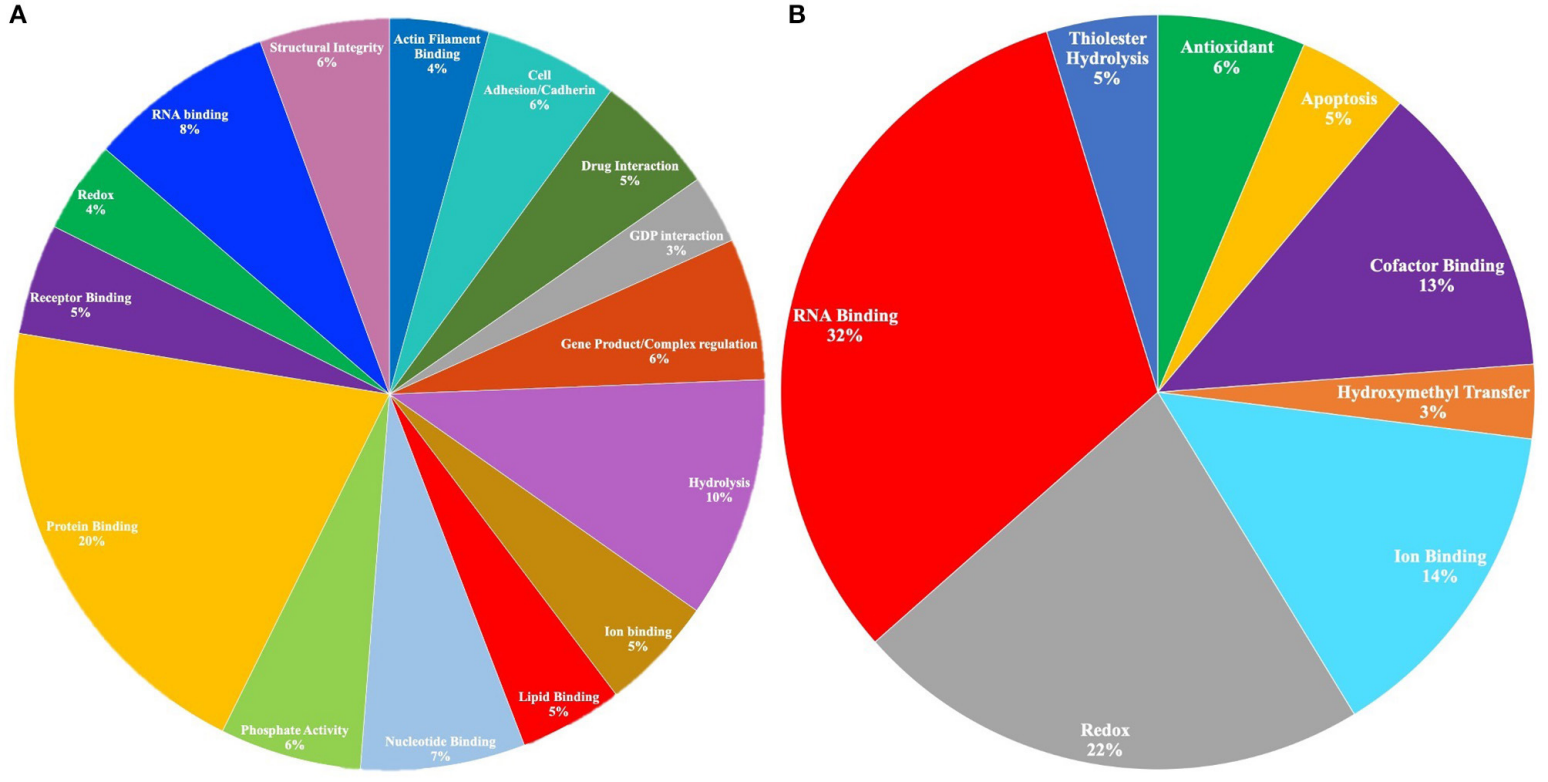
**(A)** Gene Ontology (GO) molecular functions of proteins with decreased abundance in fragile X-associated tremor/ataxia syndrome (FXTAS) (*p* < 0.05). A total of 301 proteins were submitted in the original query, and 97 proteins were associated with GO molecular functions. Only the functions associated with >1% of the proteins in the query are depicted in the chart. **(B)** GO molecular functions of proteins with increased abundance in FXTAS (*p* < 0.05). A total of 121 proteins were submitted in the original query, and 10 proteins were associated with GO molecular functions.

To elucidate cellular and biological significance of our DAPs, we used GSEA to perform GO analysis (Subramanian et al., 2005) and identify enriched GO terms associated with FXTAS DAPs (raw *p* < 0.05). Of the 303 proteins with decreased abundance, 232 proteins were associated with annotated functions/ontologies. The predominant functions of proteins with decreased abundance include protein binding (16%), hydrolysis (8%), RNA binding (6%), nucleotide binding (6%), cell adhesion (5%), gene regulation (5%), phosphate activity (5%), drug interaction (4%), structural integrity (4%), lipid binding (4%), and actin filament binding (3%) (Figure 2A). Of the 122 proteins with increased abundance, 47 proteins were associated with ontologies/functions. The predominant functions of the proteins with increased abundance include RNA binding (32%), redox (22%), iron binding (14%), cofactor binding (13%), antioxidant activity (6%), and apoptosis (5%) (Figure 2B).

### Fragile X Mental Retardation Protein-Associated mRNAs

**Figure 3:**
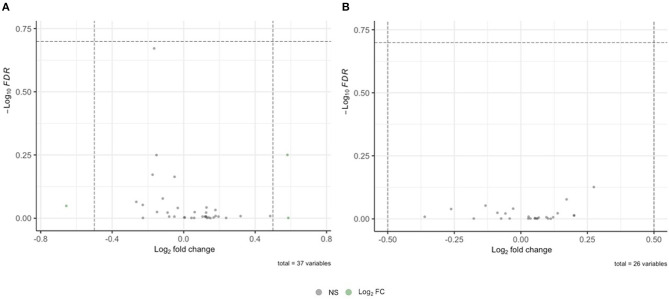
Volcano plot of mRNA and proteins associated with fragile X mental retardation protein (FMRP) in fragile X-associated tremor/ataxia syndrome (FXTAS) cortexes. **(A)** No significant differences were found in proteins derived from FMRP-associated mRNA. **(B)** No significant differences were found in proteins derived from FMRP-associated proteins.

To determine whether mRNA species known to associate with fragile X mental retardation protein (FMRP) (Pasciuto and Bagni, 2014b) are differentially expressed as proteins in FXTAS cortexes, the list of known FMRP-associated mRNAs was compared to the FXTAS proteome. Of the 54 mRNAs known to associate with FMRP, 37 (69%) were identified by MS in the FXTAS proteome (Table 3). Ras Homolog Family Member A (RHOA) protein was 11% decreased in FXTAS cortexes (*p* = 0.001, FDR = 0.21). Other nearly significant DAPs included potassium voltage-gated channel subfamily C member 1 (KCNC1) with 50% increased abundance and Ras-related C3 botulinum toxin substrate 1 (RAC1) and catenin beta-1 (CTNNB1), each with 12% decreased abundance (*p* = 0.021, FDR = 0.6). Distribution of fold change and FDR values of all 37 proteins are represented as volcano plots in Figure 3. While some of these proteins were differentially expressed at *p* < 0.05, none achieved significance of FDR < 0.2.

**Table 3:** Proteins translated from FMRP-associated mRNA in FXTAS cortex.

**Protein (gene symbol)**	**Log2FC**	**Age-adjusted raw *p*-value**	**Age-adjusted FDR**
RHOA	−0.165	0	0.213
KCNC1	0.581	0.004	0.562
CTNNB1	−0.152	0.124	0.563
RAC1	−0.174	0.008	0.673
DLGAP4	−0.051	0.744	0.686
AATK	−0.117	0.762	0.836
SPEN	−0.265	0.247	0.862
FMR1	−0.229	0.07	0.886
PLP1	−0.657	0.01	0.894
ARHGEF12	0.128	0.34	0.907
PPP2CA	−0.033	0.543	0.911
VDAC1	0.178	0.019	0.928
DAG1	−0.149	0.197	0.945
EEF2	0.062	0.428	0.946
PCDH10	0.126	0.523	0.95
PKP4	−0.09	0.135	0.95
APP	0.485	0.189	0.98
CAMK2A	0.179	0.053	0.98
KCND2	0.319	0.473	0.981
ARC	−0.052	0.872	0.985
DLG4	0.12	0.329	0.985
FUS	0.124	0.062	0.985
HNRNPA2B1	0.195	0.118	0.985
MAP1B	−0.081	0.373	0.985
OPHN1	0.126	0.466	0.985
PTPN5	0.106	0.613	0.985
AP2B1	0.14	0.109	0.993
MAP2	0.006	0.978	0.993
NLGN2	0.168	0.188	0.993
PIK3CB	0.003	0.975	0.993
ALDOA	0.044	0.28	0.997
APC	0.059	0.695	0.997
GABRB1	0.238	0.18	0.997
GABRD	0.586	0.11	0.997
MBP	−0.228	0.401	0.997
SOD1	0.131	0.085	0.997
PCLO	0.15	0.219	0.999

*FMRP, fragile X mental retardation protein; FXTAS, fragile X-associated tremor/ataxia syndrome*.

### Fragile X Mental Retardation Protein-Associated Proteins

We also analyzed proteins known to associate with FMRP (Pasciuto and Bagni, 2014a) to determine whether any are differentially expressed in FXTAS cortexes. Of the 53 proteins known to associate with FMRP, 25 were identified by MS in the FXTAS proteome (Table 4). None of the 25 FMRP-associated proteins were significantly differentially abundant in FXTAS cortexes at FDR < 0.2.

**Table 4:** FMRP-associated proteins in FXTAS cortexes.

**Protein (gene symbol)**	**Log2FC**	**Age-adjusted raw *p*-value**	**Age-adjusted FDR**
TIA1	0.274	0.067	0.748
CAPRIN1	0.172	0.145	0.837
FXR2	−0.133	0.188	0.886
KIF1B	−0.029	0.248	0.911
STAU1	−0.262	0.262	0.914
HABP4	−0.088	0.347	0.946
DDX5	0.138	0.352	0.95
FXR1	−0.058	0.387	0.952
PURA	0.2	0.45	0.969
PURA	0.2	0.45	0.969
RPL5	0.029	0.503	0.98
YBX1	−0.361	0.525	0.981
MYO5A	0.121	0.595	0.985
RALY	0.096	0.562	0.985
AGO1	0.067	0.696	0.988
CYFIP2	0.053	0.76	0.993
APC	0.059	0.933	0.997
CYFIP1	0.028	0.877	0.997
EIF4E	0.062	0.972	0.997
EIF5	−0.074	0.861	0.997
KIF5A	−0.042	0.849	0.997
NUFIP2	−0.177	0.954	0.997
PAK1	0.053	0.861	0.997
RPL8	0.102	0.94	0.997
RPLP0	0.036	0.9	0.997

*FMRP, fragile X mental retardation protein; FXTAS, fragile X-associated tremor/ataxia syndrome*.

### Calcium-Associated Proteins

**Figure 4:**
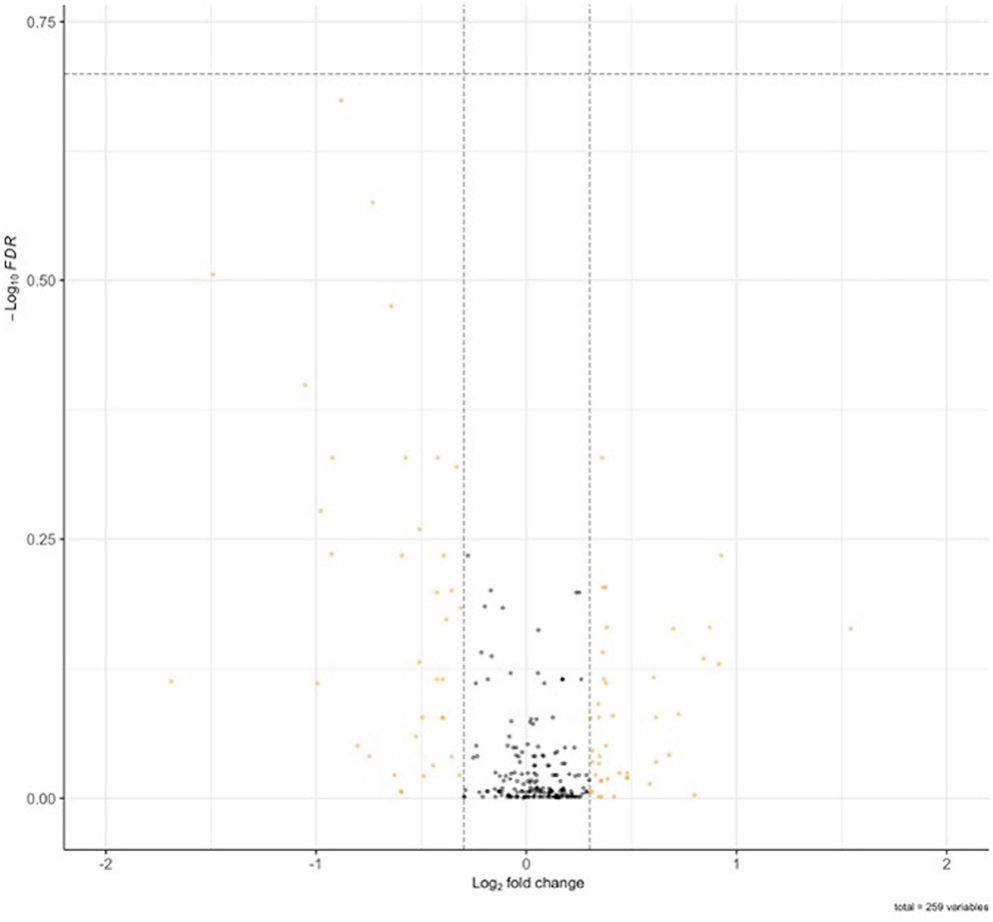
Volcano plot of Ca^2+^ associated proteins in fragile X-associated tremor/ataxia syndrome (FXTAS) cortexes. No significant differences in Ca^2+^ associated proteins were identified in FXTAS cortexes.

Of the 716 proteins associated with calcium ion binding [GO:0005509 GO_Calcium_Ion_Binding] and calcium-associated proteins associated with FXTAS [*TPCN1, TPCN2, ORAI1, ORAI2, ORAI3, CACNA1D, CACNA1F, CACNA1A, CACNA1B, CACNA1E, CACNA1G, CACNA1H, CACNA1I*, and *P2RX1-7*] (Rovozzo et al., 2016), 262 (37%) were identified by MS in FXTAS cortexes. Although none of the proteins demonstrated significantly different abundance in FXTAS cortexes at FDR < 0.2 (Figure 4), 28 proteins were significant before FDR correction (raw *p* < 0.05), including two proteins, versican proteoglycan (VCAN; FDR = 0.21) and desmoglein-1 (DSG1; FDR = 0.26), which demonstrated nearly significant decreased abundance (Table 5). Aside from calcium-ion binding, these proteins function as extracellular matrix (ECM) cell–cell adhesion, plasma membrane/cytosolic trafficking, and proliferative signaling.

**Table 5:** Calcium-binding associated DAPs (*p* < 0.05).

**Protein (gene symbol)**	**Log2FC**	**Age-adjusted raw *p*-value**	**Age-adjusted FDR**
VCAN	−0.881	0.001	0.212
DSG1	−0.732	0.002	0.266
EFEMP1	−1.491	0.003	0.312
CDH20	−0.644	0.003	0.335
CLEC3B	−1.053	0.005	0.399
CDH11	−0.423	0.011	0.469
EFHD2	0.36	0.01	0.469
EPS15	−0.576	0.01	0.469
S100A16	−0.923	0.009	0.469
SPARCL1	−0.333	0.012	0.479
S100A13	−0.979	0.017	0.528
NID1	−0.509	0.019	0.55
PCDHGC3	−0.927	0.023	0.581
C1R	0.927	0.028	0.583
HRNR	−0.28	0.026	0.583
PLS3	−0.394	0.027	0.583
PLSCR4	−0.593	0.028	0.583
EPDR1	0.365	0.036	0.626
SLC25A13	0.376	0.037	0.626
LCP1	−0.171	0.038	0.63
PLCD1	−0.357	0.037	0.63
PLS1	−0.427	0.04	0.633
SNCA	0.251	0.04	0.633
SNCB	0.237	0.04	0.633
TTYH1	−0.199	0.043	0.653
CDH2	−0.112	0.044	0.655
CDH8	−0.314	0.044	0.655
GSN	−0.382	0.049	0.672

*DAP, differentially abundant protein*.

### Comparison to Alzheimer Disease and Parkinson Disease

**Figure 5:**
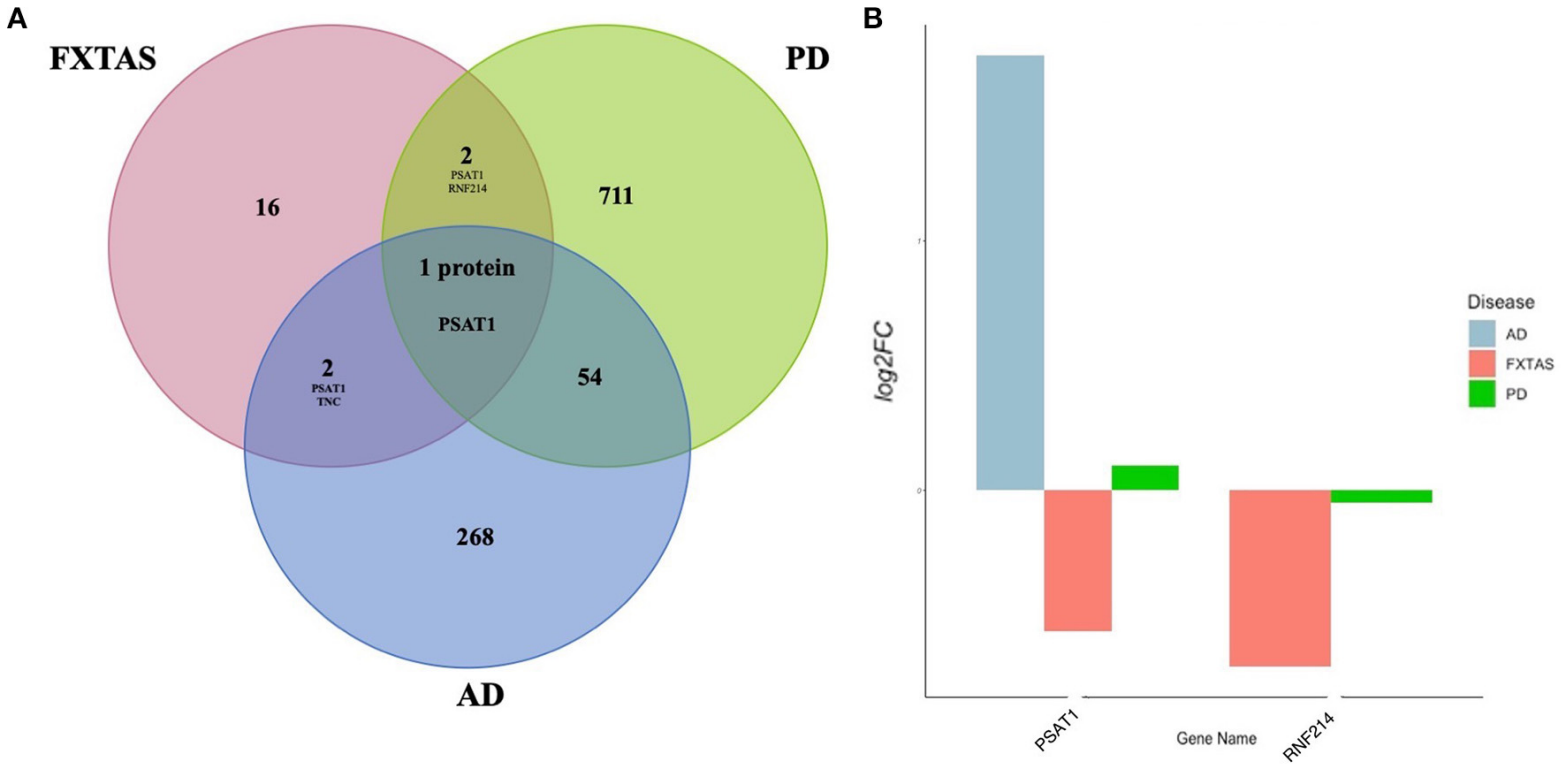
Shared differentially abundant proteins (DAPs) between neurodegenerative disorders. **(A)** Venn diagram of significant [false discovery rate (FDR) < 0.2] DAPs in fragile X-associated tremor/ataxia syndrome (FXTAS), Alzheimer's Disease (AD), and Parkinson's Disease (PD). **(B)** Log2 fold change values of proteins shared between FXTAS, PD, and AD.

Overlapping DAPs in FXTAS, Alzheimer disease (AD), and Parkinson disease (PD) were determined using the proteins described in previous proteomic analysis of PD (FDR < 0.2) reported by Dumitriu et al. (2016) and of AD reported by Hondius et al. (2016). For comparison, the FXTAS DAPs (determined by FDR < 0.2), PD DAPs (FDR < 0.2) listed in Dumitriu et al. (2016), and the AD DAPs (FDR < 0.05) listed in Hondius et al. (2016) were compared. The complete list of shared DAPs is described in Table 6. Of the shared proteins, phosphoserine aminotransferase 1 (PSAT1) and TNC (tenascin-C) were shared between AD and FXTAS, ring finger protein 214 (RNF214) was shared between PD and FXTAS, and only PSAT1 was shared between all three neurodegenerative diseases (Figure 5). Of these three proteins, all three proteins demonstrated decreased abundance in FXTAS.

**Table 6:** Comparison of FXTAS, PD, and AD proteomes^*a*^.

**Protein (gene symbol)**	**FXTAS**	**AD**	**PD**	**FXTAS Log_**2**_FC**	**FXTAS age-adjusted FDR**
PSAT1	+	+	+	−0.564	0.056
RNF214	+	ND	+	−0.564	0.134
TNC	+	+	ND	−2.858	0.056

a *AD, Alzheimer disease; PD, Parkinson disease; FC, fold change; FXTAS, fragile X-associated tremor/ataxia syndrome; PSAT1, phosphoserine aminotransferase 1; RNF214, ring finger protein 214; TNC, tenascin-C*.

## Discussion

### Fragile X-Associated Tremor/Ataxia Syndrome Proteome

A striking feature of the current proteomic analysis is that the FXTAS proteome demonstrates differential abundance compared to the proteome of the control brains of only a small number of proteins compared to that of controls. Furthermore, the majority of these DAPs were biased toward decreased abundance, of transcriptional downregulation, and/or protein degradation in FXTAS. However, the absence of any substantial shift in protein abundance for the FXTAS proteome does not rule out more substantive shifts in the phosphoproteome and proteomes of other posttranslational modifications (PTMs), which are not identified in the current analysis.

### Increased Small Ubiquitin-Like Modifier 1

Consistent with the studies of FXTAS inclusions by Ma et al. (2019), we observed modest, but significantly increased abundances of small ubiquitin-like modifier 1 (SUMO1)/SUMO1P1 and SUMO2 proteins in FXTAS cortex (50% increase in SUMO1 protein; 20% increase in SUMO2). SUMO1 is a member of the ubiquitin protein modifier super-family and forms a posttranslational, reversible bond to lysine residues on target proteins (Wilson and Heaton, 2008; Matsuzaki et al., 2015). Unlike ubiquitin modifications, SUMO proteins are involved in the regulation of a variety of cellular processes such as nuclear transport, transcriptional regulation, apoptosis, and protein stability (Zhang et al., 2016). Growing evidence suggests that SUMOylation is a critical PTM in neuronal development and function, as well as in neurodegenerative disease and ischemic injury (Krumova and Weishaupt, 2013; Lee et al., 2013; Matsuzaki et al., 2015; Zhang et al., 2016). Recently, Khayachi et al. (2018) demonstrated that metabotropic glutamate receptor type 5 (mGluR5) activation induces SUMOylation of FMRP in neurons and subsequent dissociation of FMRP from dendritic RNA granules (Khayachi et al., 2018; Tang et al., 2018), indicating a critical role for SUMOylation in the regulation of mGluR5-mediated calcium signaling, spine density, and FMRP–RNA interactions. Furthermore, SUMOylated proteins were found to accumulate in insoluble inclusions in response to proteasomal inhibition, suggesting additional roles of SUMOs in protein aggregation and response to misfolded proteins (Tatham et al., 2011). An altered SUMO response possibly precedes the characteristic hallmarks of cellular dysfunction in PM carriers, which include downregulated signaling and impaired calcium signaling, as well as disturbed mitochondrial, endoplasmic reticulum (ER), and mGluR1/5 function. Reduced function of mGluR5 receptors in end-stage FXTAS (Pretto et al., 2014) and increased accumulation of SUMO-rich proteins in inclusions suggest a significant role of SUMO in protein processing and signaling in the progression of cellular dysregulation in FXTAS.

Recent studies provided evidence for the contribution of SUMOylation to misfolded protein granules/inclusions (Tatham et al., 2011) and regulation of synaptic function (Anderson et al., 2017). While increased SUMO abundance is consistent across both FXTAS inclusions and whole-cell cortex proteomes, further studies are needed to determine if the elevated neuronal cell SUMO abundance is the result of increased SUMO-conjugated proteins or free SUMO. As PTMs of FMRP are dependent on mGluR5 activation (Westmark and Malter, 2007), the reduction in mGluR5 receptors in FXTAS neurons may be responsible for altered FMRP-PTMs and consequentially reduced FMRP-mediated clearance of clearance of RNA granules, spine density and maturation, as well as increased excitotoxicity of the remaining mGluR5s. Altered downstream regulation of mGluR5-mediated Ca^2+^ signaling may explain the relationship between dysregulated intracellular Ca^2+^ signaling present in FXTAS neurons as well as the related loss of synaptic plasticity. Additional research is needed to explore the mGluR5 SUMO relationship and determine if mGluR5 inhibitors used for AD, such as memantine HCl (Namenda), 2-chloro-4-((2,5-dimethyl-1-(4-(trifluoromethoxy)phenyl)-1H-imidazol-4-yl)ethynyl)pyridine (CTEP), or basimglurant, may similarly ameliorate FXTAS-related cognitive decline and pathology. However, a preliminary trial of memantine HCl was not found to improve tremor, ataxia, or executive function (Seritan et al., 2014), although it did improve event-related potential (ERP) studies of language processing and attention (Yang et al., 2014, 2016).

Abnormal ubiquitin-proteasome system (UPS) function has been identified in many neurodegenerative diseases (Zheng et al., 2016), leading to toxic protein-rich inclusion indicators of disease. As SUMO proteins are responsible for both dynamic regulation of proteins as well as cross-communication with the UPS system (Wilson and Heaton, 2008), differential abundance of SUMOs may represent dysregulation of the UPS in FXTAS as well. Further research on the profile of SUMO proteins and related PTMs should be explored to determine if increased SUMOs in FXTAS patients are representative of proteasomal dysregulation as well.

### Tenascin-C

**Figure 6:**
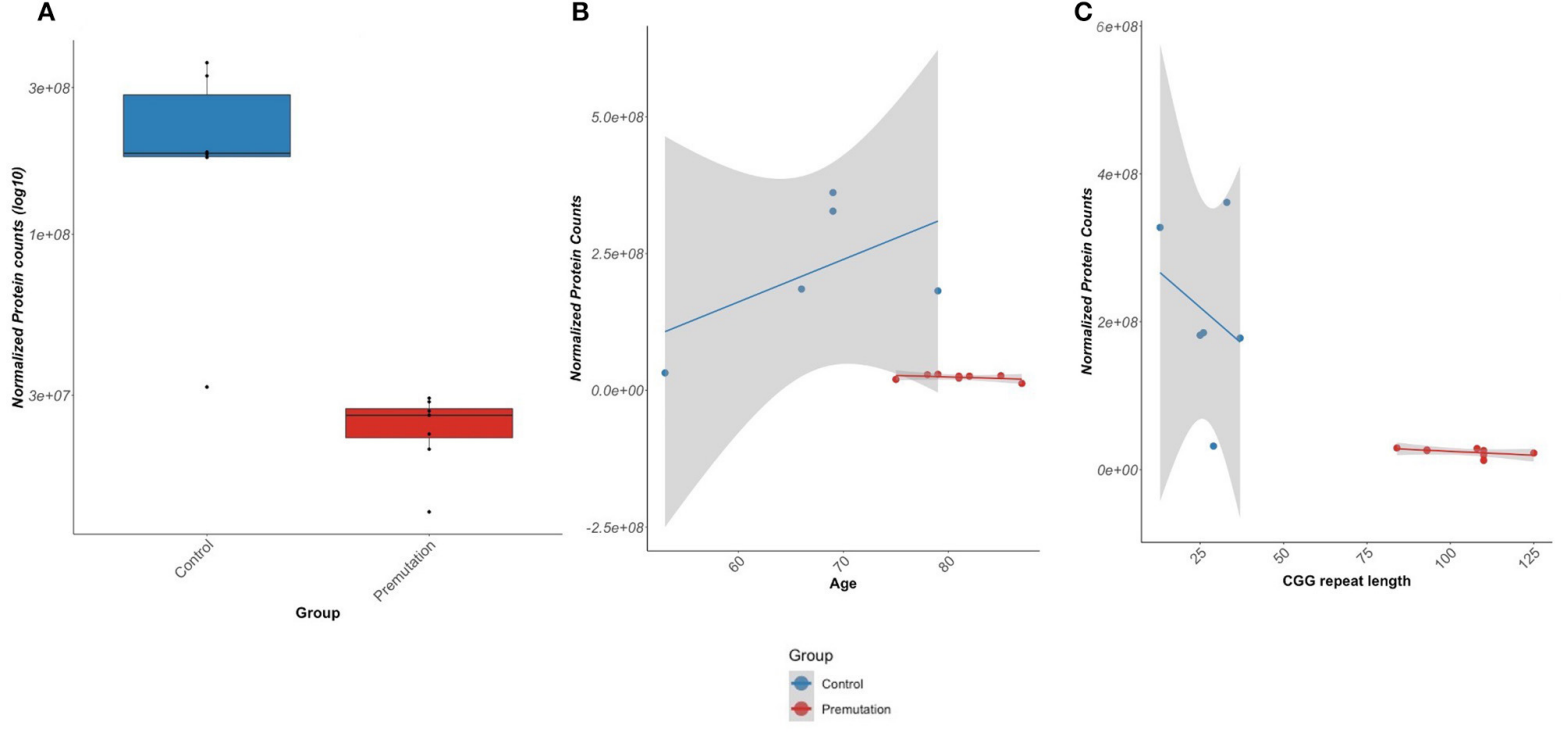
Tenascin-C (TNC) protein expression. **(A)** TNC protein demonstrates significantly lower abundance in fragile X-associated tremor/ataxia syndrome (FXTAS) than unaffected control patients [false discovery rate (FDR) = 0.56]. **(B)** TNC protein abundance is significantly lower in FXTAS than unaffected controls, independent of age. **(C)** TNC protein abundance is lower in FXTAS than unaffected controls, with a slight dependence on CGG repeat length.

Another observation of the current study is the significant decrease in abundance of TNC in the FXTAS cases. TNC is an ECM protein responsible for modulating several cellular functions, including cell adhesion, neurogenesis/axonal guidance, and mechanical stress (Midwood and Orend, 2009; Midwood et al., 2016; Wiemann et al., 2019). While TNC is highly expressed in neurons during development, the protein is indicative of growth and repair processes in adult tissue at sites of trauma, inflammation, and tumor development (Udalova et al., 2011). We observed a significant, age-independent loss of both TNC protein and mRNA expression in FXTAS cortexes (Figure 6), as well as downregulation of a number of ECM and cell–cell adhesion-associated proteins. The observed decrease of TNC protein in FXTAS is unexpected and highlights a distinction between the FXTAS proteome and those of other neurodegenerative diseases, in which increased TNC protein abundance often correlates with an inflammatory response and initiation of other neurodegenerative diseases (Xie et al., 2013; Wiemann et al., 2019).

Increased *TNC* gene expression (increased mRNA abundance) has also been implicated in response to traumatic brain injury and subsequent activation of the inflammatory response pathway (Liu et al., 2018; Dai et al., 2019). However, *TNC* gene silencing after subarachnoid hemorrhage (SAH) was found to alleviate neuronal inflammation and apoptosis *via* phosphoinositide-3-kinase (PI3K)/Akt/nuclear factor (NF)-κB pathway activation and has been highlighted as a potential therapeutic target to prevent neuronal cell death after injury (Shiba et al., 2014; Liu et al., 2018). Thus, loss of TNC protein may be consistent with a compensatory cellular response in FXTAS, and elucidation of *TNC* gene expression may provide insight into how this response is regulated. *TNC* transcription is positively regulated primarily by transforming growth factor (TGF)-β and fibroblast growth factor 2 (FGF2) in astrocytes (Chiovaro et al., 2015). Although *TGF-*β mRNA was not significantly differentially expressed in FXTAS (unpublished), TGF*-*β protein trended upward [not significant (NS)] in our FXTAS cortexes. FGF2 protein was not identified in the MS dataset but found to be nearly significant in mRNA analysis (Table 7), with a 2-fold decrease in expression in FXTAS cortexes. *FGF-2* mRNA expression was also found to increase in degenerating neurons and correlate with microglial phagocytosis of debris (Noda et al., 2014); therefore, downregulated *FGF-2* in FXTAS may contribute to increased debris accumulation. Downregulation of this microglia-mediated phagocytosis, inflammatory, and apoptotic response suggests a distinct lack of response signaling present in most other neurodegenerative disorders. The nearly complete loss of *TNC* mRNA and TNC protein expression in FXTAS cortexes suggests a failure in cell injury response and survival pathways and is indicative of impaired pathway regulation.

**Table 7:** mRNA expression of related proteins in FXTAS.

**Gene name**	**Log2FC**	**Raw p Value**	**FDR**
S100A13	−0.819661	2.51E-06	0.00824748
EFEMP1	−1.0957956	0.00786927	0.66824325
DEK	0.24087646	0.00867009	0.67817542
SPARC	−0.4185093	0.02028034	0.82975921
FGF2	−0.6323628	0.04052438	0.92959458
TGFB1	−0.1773479	0.82890012	0.99910395
TNC	−0.4960031	0.61936433	0.99910395
VCAN	−0.035502	0.86615008	0.99910395
EPS15	−0.1631534	0.10277535	0.99910395
S100A16	−0.3955453	0.25492682	0.99910395
PLSCR4	−0.5044934	0.12931283	0.99910395

*FDR, false discovery rate; FXTAS, fragile X-associated tremor/ataxia syndrome; TNC, tenascin-C*.

### Cluster of Differentiation 38

Cluster of differentiation 38 (CD38) protein, a pro-inflammatory enzyme responsible for degradation of nicotinic acid dinucleotide (NAD) and regulation of calcium-dependent myeloid-derived inflammatory cells (Blacher et al., 2019), was significantly less abundant in FXTAS cortexes. While CD38 protein abundance often increases with age and in other neurodegenerative diseases such as AD and PD (Dumitriu et al., 2016; Blacher et al., 2019; Guerreiro et al., 2020), the significant reduction of this pro-inflammatory protein is consistent with a non-inflammatory-mediated primary pathology in FXTAS. CD38 knockout models demonstrated altered microglial response and reduced pro-inflammatory cytokine secretion (Guerreiro et al., 2020). Due to the typical age-associated increase in CD38 expression, the lack of CD38 protein in FXTAS may not be due to direct downregulation but rather impaired initiation of this age-related response pathway or additional compensatory mechanisms.

### Cell Adhesion, Extracellular Matrix, and Actin Cytoskeleton

Many ECM, adhesion-interacting, and cytoskeletal proteins were among the most significant DAPs, including TNC, VCAN, CD44, and plectin (PLEC) (Li et al., 2019). While TNC, VCAN, CD44, and PLEC proteins were increased in AD patients in association with inflammation and synaptic plasticity (Hondius et al., 2016), these proteins were decreased in FXTAS cortexes, indicative of a unique and opposite regulation of this shared pathway.

Ontology analysis of the proteins with decreased abundance demonstrated strong association with cell–cell adhesion pathways, suggesting that these intercellular junctions may be lost as potential precursors to apoptosis in FXTAS neuronal cells. Altered abundance of cell–cell adhesion-related proteins in PD and AD is indicative of a mechanistic role in neurodegenerative progression (Ramanan and Saykin, 2013). This pathway may contribute to the mechanisms preceding neuronal death through initial degradation of cell–cell junctions in the autophagic neurodegenerative response. Network analysis of AD at each stage of disease progression demonstrated dysregulation in actin-based cytoskeletal and cell–cell junction regulatory proteins in the initial stages, while the intermediate stages demonstrated impaired mitochondrial functions and redox signaling imbalance, and impaired pre-mRNA splicing and RNA stability in the advanced disease stages (Li et al., 2019). Identification of dysregulated cell–cell junctions may represent the cellular phenotype of neuronal cells initiating neurodegeneration, while dysregulation of mRNA splicing and stability may represent the population of cells in late-stage neurodegeneration. As our patient-derived samples were exclusively from late-stage FXTAS cortexes, the proteomic profile is representative of advanced disease progression. Additionally, the observed FXTAS proteomic shifts may only capture cells initiating or progressing toward neurodegeneration, while already degenerated cells would be unlikely to retain intact proteins for proteomic measurement.

### Proteomic Profile of Calcium Dysfunction in Fragile X-Associated Tremor/Ataxia Syndrome

Calcium dysregulation has been observed in both hippocampal neurons from neonatal PM mice and PM patient-derived human dermal fibroblasts (Cao et al., 2013; Hagerman and Hagerman, 2015; Robin et al., 2017). PM neuronal cells demonstrate reduced expression of glutamate (Glu) transporters and the mGluR5 receptor, both of which are key mediators of calcium signaling (Cao et al., 2013; Pretto et al., 2014). Attenuated Glu signaling due to decreased mGluR5 availability is associated with increased excitotoxicity in many neurodegenerative disorders (Lewerenz and Maher, 2015; Crabbé et al., 2019) and may contribute to calcium dysfunction as well as downstream cdk5-ATM dysregulation in FXTAS.

We observed decreased abundance of the Ca^2+^ associated proteins VCAN, EGF containing fibulin extracellular matrix protein 1 (EFEMP1), epidermal growth factor receptor pathway substrate 15 (EPS15), S100 calcium binding protein A1 (S100A13), S100 Calcium Binding Protein A16 (S100A16), Cadherin 20 (CDH20), secreted protein acidic and cysteine rich (SPARC), Fibulin 1 (FBLN1), and phospholipid scramblase 4 (PLSCR4). Of these Ca^2+^-associated proteins, VCAN, EFEMP1, EPS15, S100A16, S100A13, SPARC, and PLSCR4 were also downregulated as mRNA (Table 7). While Ca^2+^ dysregulation is a common hallmark of neurodegenerative diseases, Ca^2+^-associated proteins commonly present with increased abundance in neurodegenerative diseases (Hondius et al., 2016; Bereczki et al., 2018). Furthermore, downregulation of these species as both mRNA and protein suggests transcriptional dysregulation of Ca^2+^, and upstream regulation of Ca^2+^ homeostasis is a potential mechanism of pathology. These observations are consistent with the cytosolic Ca^2+^ dysregulation observed in the murine model (Robin et al., 2017).

### Potential Biomarkers

DAPs, particularly those detected in cerebrospinal fluid (CSF) and serum at early stages of FXTAS, may serve as an early indicator of FXTAS progression and potential measurement of therapeutic efficacy. For example, TNC has been identified in CSF and serum after aneurysmal subarachnoid hemorrhage (Suzuki et al., 2010, 2011) and may serve as a potential biomarker for FXTAS. Further proteomic analysis of FXTAS CSF and/or serum in comparison to the cortex DAP biomarker candidates should be performed to determine if the FXTAS-associated loss of TNC expression is systemic throughout the nervous system and may serve as a potential biomarker for disease.

### FMRpolyG

We did not detect FMRpolyG in any of the FXTAS or control brains used for the current study, despite our detection of the peptide in FMRpolyG peptide controls and our ability to detect expanded CGG-repeat FMRpolyG-GFP expressed in cultured human neural (SK) cells (Supplementary Figure 1). Our results are consistent with the observations of Ma et al. (2019) who observed only trace amounts of the peptide in FXTAS inclusions but no peptide in whole nuclear preps for the same cases. Moreover, we are unaware of any other study of CNS tissue that has identified/quantified FMRpolyG in human brain tissue other than by immunostaining of inclusions. This suggests that endogenous FMRpolyG is not abundantly expressed in CNS tissues and thus may not be present in sufficient quantities to initiate FXTAS neuropathology. Further studies aimed at quantifying FMRpolyG levels should therefore be undertaken in the context of models proposed for its role in neurodegeneration.

### Correlation Between Differentially Abundant Proteins and Fragile X-Associated Tremor/Ataxia Syndrome-Associated Dysfunction, mRNA, and Proteins

Surprisingly, we did not identify differential abundance of mitochondrial proteins or those associated with mitochondrial dysfunction in FXTAS cortexes. Despite the evidence demonstrating altered mitochondrial dysfunction and decreased mitochondrial protein expression in PM subjects (Ross-Inta et al., 2010; Napoli et al., 2016, 2018), dysfunction was not found in association with our DAPs in late-stage FXTAS. This may be the result of late-stage disease progression and the overall cellular abundance of proteins outweighing those specifically associated with mitochondria.

We also did not detect significant changes in the abundance of FMRP-associated mRNAs or proteins. The lack of significant differential abundance of this class of proteins may simply be due to the lack of significant variation in FMRP abundance within the FXTAS cortex samples.

## Conclusions

MS analysis of male human cortex detected differential abundance of at least 16 proteins (FDR < 0.2) in late-stage FXTAS patients compared to controls. Several functional groups demonstrated decreased abundance, such as cell–cell adhesion/ECM, stress and cellular response, calcium signaling, and molecule metabolism systems. Increased abundance of proteins associated with transcriptional regulation, RNA binding, PI3K/Akt/mammalian target of rapamycin (mTOR) signaling, and SUMO regulation suggest dysregulation of pretranscriptional and posttranslational processes in FXTAS preceding neuronal death. Furthermore, many DAPs shared among other neurodegenerative disorders displayed opposite directional abundance in FXTAS when compared to the same proteins in AD and PD. This directional difference suggests potential shared degenerative pathways but differences in response mechanisms in late-stage FXTAS.

Further research on the upstream regulation of these proteins, presence of PTM modifications, subcellular localization, and correlation with mRNA levels should be addressed. Additional brain cell type-specific isolation and analysis would provide further insight into the differential function specialized to glia, astrocytes, oligodendrocytes, microglia, and neurons, as cell type-specific changes were identified in other neurodegenerative disorders (Cao et al., 2013; Pretto et al., 2014; Johnson et al., 2018; Wilson and Nairn, 2018; Dixit et al., 2019). Surprisingly, native FMRPolyG was not identified in FXTAS cortexes. Inflammatory-associated proteins were also significantly less abundant and may suggest an impaired inflammatory response or non-inflammatory pathway unique to FXTAS pathology when compared to other neurodegenerative diseases. These DAPs provide unique insight into mechanisms of FXTAS pathology, potential therapeutic targets, and candidate biomarkers for disease progression.

### Limitations

Protein and RNA degradation are inherent to *postmortem* tissue, presenting a likely reduced number of transcript and protein abundance in our patient-derived cortex samples. As input proteins do not undergo amplification before detection, certain proteins may be present below the detection limit of non-targeted MS analysis. Lack of detection of proteins of interest is not confirmation of protein absence but may be the result of very low quantity/trace amounts. Targeted protein quantification is needed to determine if proteins of interest not identified in this study are in fact present at levels below the level of detection/quantification or if they were lacking altogether. In this regard, we were not successful in detecting TNC or CD38 using Western blot for either controls or FXTAS cases. This inability to detect these proteins by Western blot likely reflects their low concentrations in adult brain tissue. In our MS analysis, TNC represented about 14 ppm of total protein abundance and CD38, about 2.5 ppm. We note that in their study of CD38 in brain tissues, Mizuguchi et al. (1995) were unable to detect CD38 by Western blot unless they first enriched for CD38 by immunoprecipitation.

Access to human cortex samples was also limited, and the small sample size of eight FXTAS subjects and six control subjects likely resulted in lower statistical power while controlling for multiple testing by FDR for high-throughput proteomic data. To account for these limitations, proteins with a raw *p* < 0.05 but within the top 7% of differentially expressed proteins were considered for molecular function analysis. The nature of sample collection also resulted in *postmortem* interval (PMI) variation between subjects and groups. While all brain sections were obtained from the cerebral cortex, future studies are warranted to characterize differential protein abundance among additional brain regions.

## Data Availability Statement

The raw data supporting the conclusions of this article will be made available by the authors, without undue reservation.

## Ethics Statement

All procedures were performed in compliance with the UCD Institutional Review Board and the 1964 Helsinki declaration, including subsequent amendments and ethical standards.

## Author Contributions

JR contributed additional data and edits upon reviewer-suggested revisions. All authors listed have made a substantial, direct and intellectual contribution to the work, and approved it for publication.

## Conflict of Interest

The authors declare that the research was conducted in the absence of any commercial or financial relationships that could be construed as a potential conflict of interest.

## Supplementary Material

Supplementary Figure 1 FMRpolyG detected by western blot. SK-N-MC cells transfected with expression plasmid produced FMRpolyG with 63 glycines fused to GFP (**FpG-GFP**, 38.5kD) or **GFP** (26.9kD) alone as a control. For each sample, 80 micrograms total protein determined by BCA assay (Pierce 23255) were run on a Criterion™ TGX Any kD™ Precast Gel (Bio-Rad 567-1124) alongside 5 microliters of Chameleon Duo Pre-Stained Protein Ladder (LI-COR 928-60000) (**Marker**) for 45 minutes at 100 mA. Proteins were transferred to a nitrocellulose membrane at 30V overnight at 4°C. The membrane was blocked in 5% BSA in TBS for 1 h at room temperature. Rabbit anti-GFP (Invitrogen A-1122, 1:5,000) and mouse anti-FMRpolyG (gift from the lab of Nicholas Charlet #8MFM2F7, 1:100) primary antibodies in blocking buffer supplemented with 0.1% Tween-20 were applied to the membrane overnight at 4°C. LI-COR IRDye® 800CW anti-rabbit IgG and IRDye® 680LT anti-mouse IgG secondary antibodies in blocking buffer supplemented with 0.1% Tween-20 were applied at 1:20,000 for 1 h at room temperature. Membrane was visualized on the LI-COR Odyssey Near-Infrared (NIR) Fluorescent Imager in the **(A)** 700-nm and **(B)** 800-nm channel. A composite image **(C)** identifies FMRpolyG-GFP as overlapping GFP and FMRpolyG signals (see arrow).
