# Corrigendum: Human Cerebral Cortex Proteome of Fragile X-Associated Tremor/Ataxia Syndrome

**DOI:** 10.3389/fmolb.2021.695407

**Published:** 2021-05-11

**Authors:** Katharine Nichole Holm, Anthony W. Herren, Sandra L. Taylor, Jamie L. Randol, Kyoungmi Kim, Glenda Espinal, Verónica Martínez-Cerdeño, Isaac N. Pessah, Randi J. Hagerman, Paul J. Hagerman

**Affiliations:** ^1^ Department of Biochemistry and Molecular Medicine, University of California Davis School of Medicine, Davis, CA, United States; ^2^ Mass Spectrometry Research Core, University of California Davis, Davis, CA, United States; ^3^ Department of Public Health Sciences, Division of Biostatistics, University of California Davis School of Medicine, Davis, CA, United States; ^4^ Medical Investigation of Neurodevelopmental Disorders Institute, University of California Davis School of Medicine, Davis, CA, United States; ^5^ Department of Pathology and Laboratory Medicine, University of California Davis School of Medicine, Davis, CA, United States; ^6^ Department of Molecular Biosciences, University of California Davis School of Veterinary Medicine, Davis, CA, United States; ^7^ Department of Pediatrics, University of California Davis School of Medicine, Davis, CA, United States

**Keywords:** FXTAS, FMR1, DIA-MS, FMRpolyG, fragile X syndrome, CD38, tenascin-c, SUMO1/2

An author name was incorrectly spelled as “Verónica Martiínez-Cerdeño.” The correct spelling is “Verónica Martínez-Cerdeño.”

The authors apologize for this error and state that this does not change the scientific conclusions of the article in any way. The original article has been updated.

