# Supplementary figures and images for: Human Cerebral Cortex Proteome of Fragile X-Associated Tremor/Ataxia Syndrome
Source: Front Mol Biosci. 2021 Jan 29;7:600840. doi: 10.3389/fmolb.2020.600840 (PMC7879451; doi:10.3389/fmolb.2020.600840)

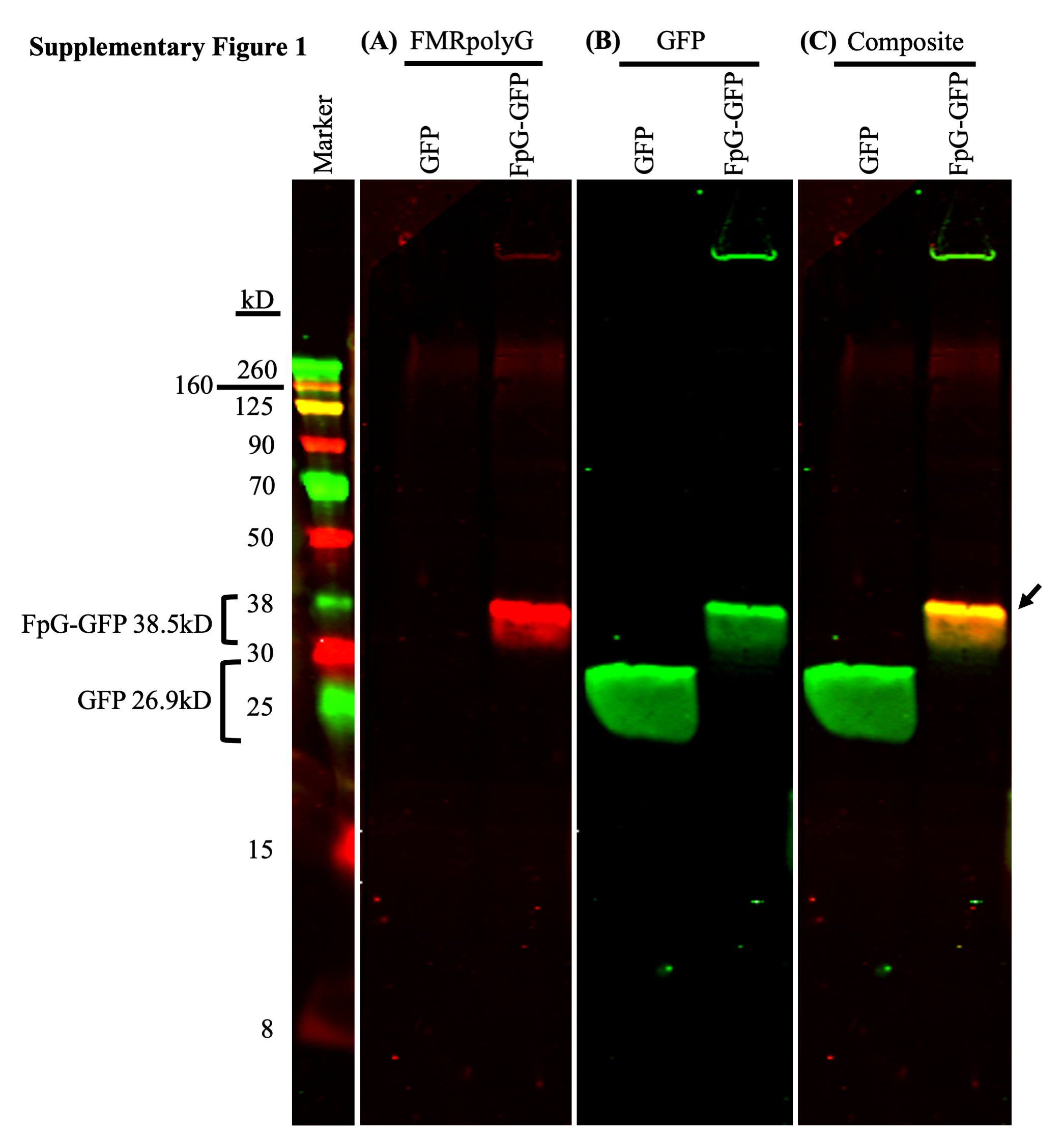

Supplement: Supplementary Figure 1 — FMRpolyG detected by western blot. SK-N-MC cells transfected with expression plasmid produced FMRpolyG with 63 glycines fused to GFP (FpG-GFP, 38.5kD) or GFP (26.9kD) alone as a control. For each sample, 80 micrograms total protein determined by BCA assay (Pierce 23255) were run on a Criterion™ TGX Any kD™ Precast Gel (Bio-Rad 567-1124) alongside 5 microliters of Chameleon Duo Pre-Stained Protein Ladder (LI-COR 928-60000) (Marker) for 45 minutes at 100 mA. Proteins were transferred to a nitrocellulose membrane at 30V overnight at 4°C. The membrane was blocked in 5% BSA in TBS for 1 h at room temperature. Rabbit anti-GFP (Invitrogen A-1122, 1:5,000) and mouse anti-FMRpolyG (gift from the lab of Nicholas Charlet #8MFM2F7, 1:100) primary antibodies in blocking buffer supplemented with 0.1% Tween-20 were applied to the membrane overnight at 4°C. LI-COR IRDye® 800CW anti-rabbit IgG and IRDye® 680LT anti-mouse IgG secondary antibodies in blocking buffer supplemented with 0.1% Tween-20 were applied at 1:20,000 for 1 h at room temperature. Membrane was visualized on the LI-COR Odyssey Near-Infrared (NIR) Fluorescent Imager in the (A) 700-nm and (B) 800-nm channel. A composite image (C) identifies FMRpolyG-GFP as overlapping GFP and FMRpolyG signals (see arrow). [file Image_1.tiff]
